# Infection of the oral cavity with SARS-CoV-2 variants: Scope of salivary diagnostics

**DOI:** 10.3389/froh.2022.1001790

**Published:** 2022-10-31

**Authors:** Parvati Iyer, Takahiro Chino, David M. Ojcius

**Affiliations:** ^1^ Department of Diagnostic Sciences, University of the Pacific, Arthur Dugoni School of Dentistry, San Francisco, CA, United States; ^2^ Department of Biomedical Sciences, University of the Pacific Arthur A. Dugoni School of Dentistry, San Francisco, CA, United States

**Keywords:** COVID-19, coronavirus, SARS-CoV-2, diagnostic, saliva

## Abstract

Coronaviruses, including SARS-CoV-2, have caused pandemics in the past two decades. The most prevalent SARS-CoV-2 variants of concern can re-infect individuals who have been previously infected with other variants or had protection from vaccines targeting the original SARS-CoV-2 variant. Given the high risk of transmission of coronavirus *via* aerosols produced during dental procedures, it is important to understand the future risk of coronavirus infection for oral health professionals and to diagnose quickly early stages of outbreaks. Testing of saliva for coronavirus may be the least invasive and most convenient method for following the outbreak at the individual and community level. This review will describe strategies for diagnosis of coronavirus in saliva.

## Introduction

The Coronavirus disease 19 (COVID-19) pandemic, caused by infection with the Severe Acute Respiratory Syndrome Coronavirus 2 (SARS-CoV-2), has killed more than 6 million individuals globally as of July 2022 (1). The symptoms of infection range from asymptomatic; to coughs, fever, and fatigue in moderate disease; to severe pulmonary pathology requiring hospitalization and ventilators. Persons with underlying co-morbidities are at a higher risk for severe disease. Though the patients with mild to moderate disease recover quickly, some report post-COVID-19 symptoms months to years after infection, even in individuals who experienced mild symptoms. For those who suffered a severe infection, it is possible for lung and cardiac function to be impaired permanently leading, to an increased risk for other complications in the future (2).

The first modern coronavirus pandemic occurred in 2002 in China and the disease was named the Severe Acute Respiratory Syndrome (SARS). In 2012, a pandemic caused by another coronavirus emerged in the middle east and was named the Middle East Respiratory Syndrome (MERS-CoV).

SARS-CoV-2 is the fifth recent coronavirus to infect humans on a wide scale, and as the mutations continue to evolve rapidly, it is crucial to understand the mechanics of prevention, genomics, and pathogenesis of the new mutations (3). The virus responsible for this pandemic has a higher rate of transmissibility and infectivity compared to the previous pandemic coronaviruses, SARS-CoV and MERS-CoV (4).

## SARS-CoV-2 mechanism of infection

SARS-CoV-2 is a single-strand positive-sense RNA virus. The SARS-CoV-2 spike (S) protein, the essential antigenic determinant of the virus (5), binds to its receptor angiotensin-converting enzyme 2 (ACE2), initiating viral entry to the host cell (6–8). The S protein consists of 1,273 amino acids and contains a signal peptide (amino acids 1–13), the S1 subunit (14–685) and the S2 subunit (686–1,273). The receptor-binding domain (RBD) in the S1 subunit recognizes ACE2, while S2 subunits play a role in membrane fusion (9). ACE2 is expressed by a variety of tissues, including pulmonary and extrapulmonary tissues (10). This accounts at least partially for extrapulmonary manifestations associated with COVID-19 (11). It has been proposed that the nasal cavity (12, 13) and oral cavity (14–17) are potential initial targets among the upper respiratory system.

SARS-CoV-2 utilizes a dual entry mechanism, cell surface and endosomal pathways, for its internalization (18–20). In the cell surface pathway, S protein can be proteolytically cleaved by host cell-derived transmembrane serine proteases (TMPRSSs). This results in the exposure of the fusion domain in the S2 subunit, which subsequently mediates viral—host cell membrane fusion (18). The clathrin-mediated endosomal pathway is carried out when the availability of TMPRSSs is limited (19). In this pathway, cathepsins play a major role in cleaving the S protein (20).

COVID-19 is primarily a disease of pulmonary tissues. However, many patients with COVID-19 also develop gastrointestinal (GI) symptoms. Given the high expression of ACE2 and the visualization of viral nucleocapsid in the GI tract (21), SARS-CoV-2 potentially enters GI cells *via* ACE2 and directly causes damage to the GI tract. In such a case, the oral cavity, in particular saliva (22), may be a potential reservoir of SARS-CoV-2 since the oral cavity and GI system are linked by constant flow of saliva. An inflammatory cytokine storm (23) and alteration of the GI microflora in response to viral infection (24, 25) have been considered as indirect factors that damage the GI tract.

SARS-CoV-2, like other respiratory viruses, can be transmitted *via* direct (physical) contact with infected individuals, indirect contact with fomites, and droplets and aerosols produced by coughing (26). In addition, the fecal-oral route has been suggested as a potential transmission route. This is based on the observation in the nonhuman primate model that intranasal or intragastric inoculation with SARS-CoV-2 resulted in both pulmonary and GI infections (27).

## Emergence of SARS-CoV-2 variants

SARS-CoV-2 continuously evolves by genetic mutations in the *S* gene. A number of SARS-CoV-2 lineages have been identified, such as Alpha (B.1.1.7), Beta (B.1.351), Delta (B.1.617.2), and Omicron (B.1.1.529) (27–30). The most prevalent SARS-CoV-2 variants of concern are currently the BA.4 and BA.5 subvariants of the Omicron variant, which can re-infect individuals who have been previously infected with other variants or had protection from vaccines targeting the original SARS-CoV-2 variant (31, 32).

Host immunity against SARS-CoV-2 is activated once the virus gains access into the host cells. Type I interferons (IFNs) play a pivotal role in both innate (33) and adaptive (34) antiviral immunity. SARS-CoV-2 can evade host immune responses by inhibition of type I IFNs, and interfering with downstream signaling molecules of Toll-like receptors (TLRs) and the JAK-STAT pathway (35–39). It has been reported that the risk of death from COVID-19 is much greater in patients with type I IFN autoantibodies (40–42).

The S protein associated with the B.1.1.7 lineage reveals several amino acid mutations. However, among them, N501Y in the RBD region accounts for at least part of the enhanced binding affinity for ACE2, enhanced virulence, and increased transmissibility (30). The B.1.351 variant expresses amino acid mutations K417N, E484K, and N501Y in RBD. This variant is reported to enter host cells more easily due to its enhanced binding affinity for ACE2 (28). It is also known to reduce the efficacy of neutralizing antibodies and the original mRNA vaccines.

The B.1.617.2 variant, first identified in India, is one of three sublineages of B.1617 and became the most predominant variant globally in late 2020 by outcompeting pre-existing lineages, such as B.1.1.7 and B.1.617.1 (43). This lineage is characterized by higher transmissibility than the wild-type Wuhan-1 D614G-bearing lineages. It is also associated with a sixfold decrease in sensitivity to convalescent antibodies and eightfold decrease in sensitivity to vaccine-elicited antibodies than the ancestral strain (44). This is partially due to the mutations L452R and T478K in RBD that play a role in increased receptor-binding affinity, infectivity, and reduced sensitivity to vaccine-elicited antibodies. The E484Q mutation in RBD is reported to augment the receptor binding affinity and reduce sensitivity to antibody (45). In addition, the P681R mutation between the S1/S2 cleavage site contributes to enhanced transmissibility (29).

The B.1.1.529 lineage (Omicron) was originally detected in South Africa in November 2021 (46). This lineage is reported to express several mutations observed in other lineages. They include N501Y, E484K, T478K, and P681R, suggesting that this lineage is potentially highly infectious and transmissible and has the ability to evade detection by the host immune system. At the same time, while being highly transmissible, the B.1.1.529 lineage has a decreased ability to infiltrate the lungs, thus the observed reduced pathogenicity (47). Accumulating evidence has elucidated that the enhanced binding of the B.1.1.529 variant to host ACE2 accounts for the enhanced transmissibility of this lineage compared with other lineages. Nonetheless, B.1.1.529 entry into lung cells is compromised since the S protein of this lineage is not cleaved efficiently by TMPRSS2. This implies that the B.1.1.529 virus relies heavily on the endosomal pathway for internalization into the host cell. Collectively, B.1.1.529 has a decreased capacity to enter the host lung cells (i.e., it is less infectious), resulting in lower replication potential than other lineages.

## Variants of concern and the oral cavity

The Centers for Disease Control (CDC) in the US has classified the variants into 3 main categories: Variant of Concern (VOC), Variant of Interest (VOI), and Variant Being Monitored (VBM). Thus, in November 2021, the Omicron variant was classified as a VOC (48, 49).

The variants have piqued the interest of researchers because of different molecular features that they exhibit in different infected populations, which in turn, has led to further studies on factors influencing the mutations of the virus. The findings might be valuable with respect to predicting vaccine efficacy against infection with future variants (50–54). One study retrospectively analyzed the emergence of the Omicron variant and determined that the epidemiology of Omicron infection was an accurate predictor of mutations in the virus, and the global trajectory was a better predictor than epidemiology at a country level. This type of understanding could help us to prepare for future pandemics (55).

**Figure 1:**
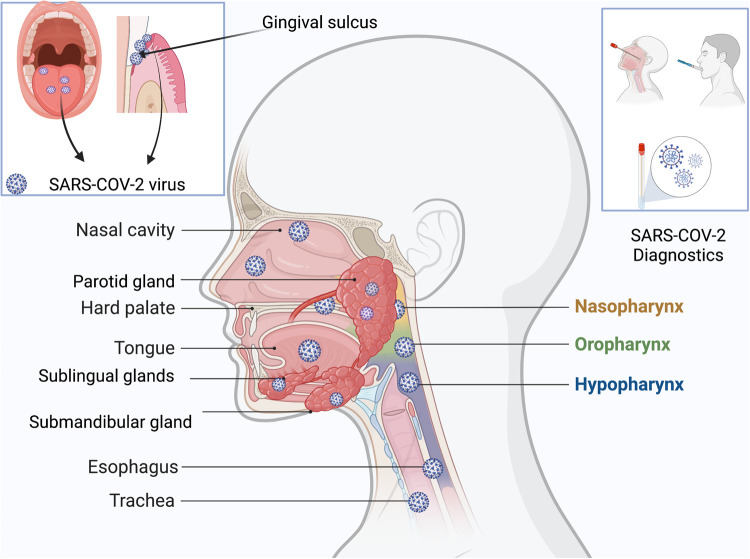
Reservoirs of SARS-CoV-2 in the head and neck regions, especially parts of the oral cavity: tongue, gingival sulci, hard palate, parotid glands, submandibular glands, sublingual glands, nasal cavity; nasopharynx, oropharynx, hypopharynx, esophagus, and trachea. Specimens for diagnostics could be derived from nasopharyngeal swabs or saliva.

Research done during earlier stages of this pandemic showed the virus's predilection for certain sites in the body, including the oral cavity, due to the presence of the ACE2 receptors in the epithelium of oral tissues. In fact, the oral cavity might be the initial site of entry for the coronavirus. The tongue, floor of the mouth, gingival sulcus, and gingival epithelium of the buccal and lingual surfaces of teeth all express ACE2 receptors (56) (Figure 1). Oral symptoms like loss of taste often precede other symptoms of the infection. Dry mouth, inflammation of gingival tissues and ulcerations have also been reported. Since a variety of viruses and other pathogens are usually present in the gingival sulcus, this site might act as a reservoir for SARS-CoV-2. The oral viral load has also been correlated with the severity of SARS-CoV-2 infection. Therefore, by reducing the viral load in the oral cavity, it may be possible to reduce the risk of transmission (14, 57, 58).

IgA appears to be the first antibody detected in response to SARS-CoV-2 infection (59) and IgA dominates SARS-CoV-2-specific antibody responses over IgG and IgM during the early stages of infection (60). Virus-specific salivary IgA and IgG have been detected in saliva up to 73 days and 9 months, respectively (60, 61). Specific serum IgA decreases 1 month after the disease onset while serum IgG is detectable up to 9 months (60, 61). Taken together, the observations suggest that SARS-CoV-2 specific salivary IgA may be a potential biomarker during the initial stage of infection. Salivary IgG response may be useful to monitor the systemic immunity to SARS-CoV-2.

An accurate and early diagnosis of the oral manifestations of the infection (62) may also contribute to minimizing progression of infection to severe disease and prevent transmission to other individuals. By analyzing risk factors for SARS-CoV-2 infection, researchers have attempted to understand and predict the progression of this infection in patients with underlying medical conditions such as diabetes or cardiovascular disease.

## Salivary diagnostics for COVID-19

Saliva has proven to be a very convenient, non-invasive source of biomarkers for many cellular and systemic reactions occurring in the body during various disease states (63–67), including COVID-19. Since SARS-CoV-2 infection is also initiated in the oral and nasal cavity, detection of the coronavirus in saliva is likely to reflect early stages of infection.

Saliva can be collected using swabs, by coughing, or by collecting it directly from the salivary glands (68, 69). One study showed that the salivary glands are a reservoir for the coronavirus (70) (Figure 1). By directly measuring the viral load in the secretions of the glands, acute infection could be assessed, and appropriate measures taken to inhibit and/or prevent transmission.

Previous studies on COVID-19 have already shown that the coronavirus could be observed in the saliva before it could be isolated from the lungs. This reinforces the view that salivary diagnostics could be used to detect and therefore to prevent transmission, especially in asymptomatic individuals (71).

While some studies discovered that impairment of the gustatory senses was an early symptom of asymptomatic patients and patients with a mild degree of infection, they also concluded that ageusia is an important and unique feature of SARS-CoV-2 infection (72, 73). The various oral manifestations of SARS-CoV-2 infection could also shed light on the interplay between ACE2 receptors, angiotensin enzyme, hyposalivation, zinc deficiency, cellular inflammation in the taste buds, and possible central nervous system degradation by the virus (74).

Once saliva was deemed to be a good diagnostic tool to diagnose early infection, researchers found that self-collected specimens of saliva from the oral cavity and fluid from the nasal swabs provided the best sensitivity and specificity compared with specimens collected through invasive methods, such as collections from the trachea and bronchiolar lavage (75, 76). In one study, saliva was shown to be positive even before nasopharyngeal swabs tested positive for the infection (77). A recent study argued that studies did not indicate clearly how their samples of saliva were obtained and questioned the conclusions (78). Another study suggested future research should consider cellular proteases to understand why the virus was able to overcome natural immune responses to the infection (79). The authors of the study postulated that a modified device to collect secretions from salivary glands directly would bypass the concern that viruses were transported from elsewhere into the saliva. The biggest challenge with salivary specimens was the quality of the salivary specimens from hospitalized patients that were thick and stringy, requiring additives before processing (80).

Oral healthcare professionals can help to identify individuals at risk by using salivary diagnostics. Though it has shown that the viral load is high in nasopharyngeal swabs and the saliva during the first week of infection, a higher viral load and increase in the age of the patients correlated with severity of disease; the accuracy of the detection of the virus in the salivary samples thus requires more investigation (81). Despite these limitations, Point of Care (POC) salivary diagnostics is still promising with respect to early detection by oral healthcare professionals. Since patients may have viral presence in their saliva and be asymptomatic, oral healthcare professionals need to take adequate precautions to prevent transmission to themselves and others by using POC diagnostics (82). This would be the best strategy to slow down spread of the infection and prevent emergence of further mutations.

The current diagnostic tests for SARS-CoV-2 infection are based on molecular detection of viral RNA *via* (polymerase chain reaction) PCR analysis, and rapid-antigen tests that detect viral protein. In general, PCR tests are more accurate, but the technique is slower than detection of viral antigen, and PCR can detect nucleic acids when the individuals are no longer infectious (83–89). However, newer molecular tests based on CRISPR nucleases can measure viral nucleic acids faster than traditional PCR tests (90).

In conclusion, regardless of the method used to detect the coronavirus, future research should be directed to predicting the trajectory of mutations in coronaviruses, the mechanisms of pathogenesis due to infection with new variants, and development of POC systems that are high in sensitivity and specificity. The hope is that early diagnosis will help to prevent or dampen future waves of SARS-CoV-2 infections.
